# Functional and spatial rewiring principles jointly regulate context-sensitive computation

**DOI:** 10.1371/journal.pcbi.1011325

**Published:** 2023-08-11

**Authors:** Jia Li, Ilias Rentzeperis, Cees van Leeuwen

**Affiliations:** 1 Brain and Cognition unit, Faculty of psychology and educational sciences, KU Leuven, Leuven, Belgium; 2 Cognitive and developmental psychology unit, Faculty of social science, University of Kaiserslautern, Kaiserslautern, Germany; University of Groningen, NETHERLANDS

## Abstract

Adaptive rewiring provides a basic principle of self-organizing connectivity in evolving neural network topology. By selectively adding connections to regions with intense signal flow and deleting underutilized connections, adaptive rewiring generates optimized brain-like, i.e. modular, small-world, and rich club connectivity structures. Besides topology, neural self-organization also follows spatial optimization principles, such as minimizing the neural wiring distance and topographic alignment of neural pathways. We simulated the interplay of these spatial principles and adaptive rewiring in evolving neural networks with weighted and directed connections. The neural traffic flow within the network is represented by the equivalent of diffusion dynamics for directed edges: consensus and advection. We observe a constructive synergy between adaptive and spatial rewiring, which contributes to network connectedness. In particular, wiring distance minimization facilitates adaptive rewiring in creating convergent-divergent units. These units support the flow of neural information and enable context-sensitive information processing in the sensory cortex and elsewhere. Convergent-divergent units consist of convergent hub nodes, which collect inputs from pools of nodes and project these signals via a densely interconnected set of intermediate nodes onto divergent hub nodes, which broadcast their output back to the network. Convergent-divergent units vary in the degree to which their intermediate nodes are isolated from the rest of the network. This degree, and hence the context-sensitivity of the network’s processing style, is parametrically determined in the evolving network model by the relative prominence of spatial versus adaptive rewiring.

## Author summary

Context-sensitivity plays an important role in neural signal processing. A type of network structure supporting context-sensitive processing is known as a convergent-divergent unit. Convergent-divergent units can give rise to sensory neurons that respond to local features but are also modulated, in their activities, by long-range contextual information. These units can arise through self-organization in neural networks according to adaptive rewiring, a principle used to optimize network structure for information processing. Two spatial rewiring principles, distance minimization and topographic alignment, are also considered important in shaping network connectivity structure, but their role in the formation of context sensitivity is unknown. We asked whether these spatial rewiring principles can facilitate adaptive rewiring in the formation of convergent-divergent units. Using weighted digraphs, we found that the proportion of rewiring based on distance minimization improves context-sensitivity, as shown by the increased robustness of the convergent-divergent units, while the subtler effects of the alignment principle depend on the shape of the topographic map that network connections align with.

## Introduction

**Fig 1:**
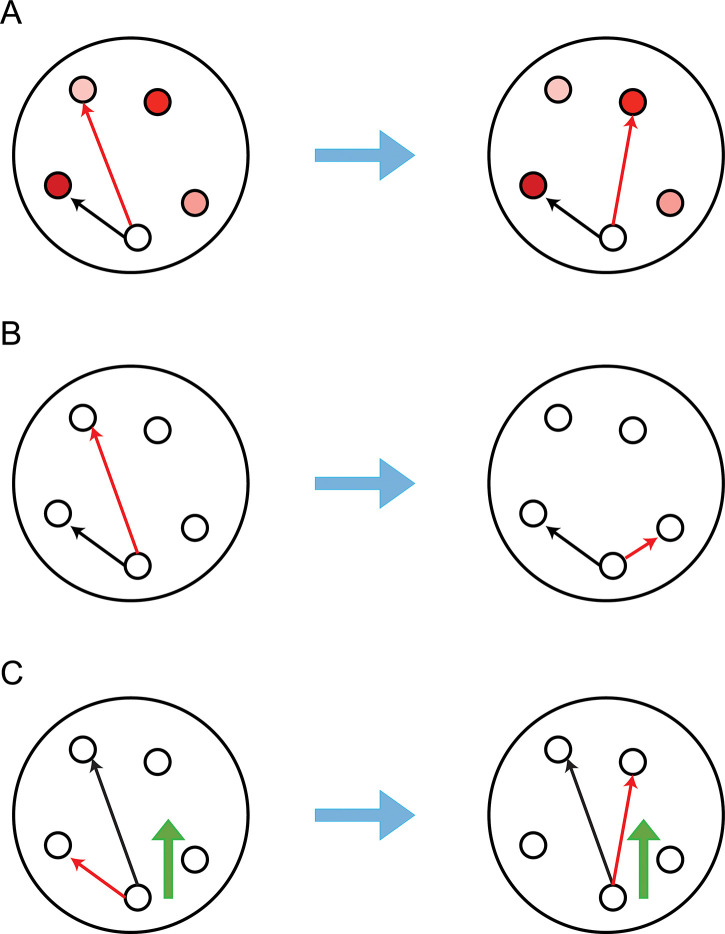
Principles of network rewiring. (A) Adaptive rewiring. The lightness of a node’s color represents the intensity of its communication with the white node. The darker the color, the more intense the communication is (B) Minimization of wiring distance. (C) Alignment to an external vector field. The red and green arrows indicate the rewiring link and the direction of the vector field respectively.

In the course of brain development, neuronal connections are constantly changing [1–3]. This continued evolution has been modeled by an elementary principle known as adaptive rewiring (Fig 1A) [4,5]. Adaptive rewiring facilitates signal processing by attaching shortcut connections to regions where neural signal traffic is intense while pruning underused connections. Adaptive rewiring is constrained within the physical space of the brain where spatial rewiring principles, such as wiring distance minimization [6] or topographic alignment of neural pathways [7], play an important role. We consider whether these spatial principles facilitate adaptive rewiring in evolving directed, weighted neural networks. In particular, we study the contribution of these spatial principles to forming convergent-divergent units: neural network structures that are pervasive in the brain at different scales [8,9].

### Adaptive and spatial rewiring principles

Adaptive rewiring generates networks with a number of complex topological features, including scale-freeness [5], small worlds [10], modularity [11,12], and the rich club effect [13]. These features reflect some of the most distinctive macroscopic characteristics of brain anatomy (for the small world, see [14]; for the rich club effect, see [15,16]).

Adaptive rewiring establishes these brain-like structure in models of oscillatory neural mass activity [5,10] as well as in neuronal-level spiking networks [17]. Spike propagation in neural networks can be represented as random walks on a graph [18], and these, in turn, can be stochastically described as graph diffusion [19]. Graph diffusion offers a particularly parsimonious account of neural activity, suitable for implementing adaptive rewiring in neural network models in a computationally efficient manner [20].

Current adaptive rewiring models have mostly been too simplified for representing the anatomical networks of the brain because they are exclusively concerned with optimizing topological features, while ignoring the spatial economy of the brain. For instance, a prominent spatial feature of the brain is that adjacent regions tend to have similar functions, and neural connections are aligned in fiber bundles and layers. This indicates that besides being adaptive, rewiring also follows certain spatial optimization principles.

One spatial principle involves minimization of wiring distance [21] by topologically connecting spatially adjacent nodes (Fig 1B). Naturally, this principle pushes for the reduction of long-range connections while establishing local connections but, when combined with adaptive rewiring, a stable, albeit sparse, proportion of long-range connections remains [22]. These long-range connections preferentially attach to hub nodes, while short-distance connections are assigned to nodes within the same topological modules. The differentiation into long- and short-range connections evolves gradually, similarly to what happens in the developing brain [23]. This results in a rewired network akin to a topographical map, a widespread functional architecture in the brain, as exemplified by the visual and somatosensory cortices.

Another spatial principle is topographic alignment (Fig 1C), whereby neural connections tend to extend either in the same direction, as the axons of pyramidal cells in the cortex, or spread or converge in a concentric fashion, as the dendrites of ganglia. A possible mechanism for alignment is that neuronal extensions develop along a vector field; either a neurochemical gradient or a traveling electrical wave field [24,25]. Propagating wave fields have been proposed to play an active role in shaping cortical maps [26]. To the extent that wave fields are homogeneous or vary smoothly across spatial regions, rewired connections that align with the direction of the wave field tend to become spatially aligned with each other. Depending on the organization of the wave field, regular topography may arise, e.g., layers as a result of a homogeneously lateral wave or ganglia as a result of a radially expanding wave [27].

In a recent study on undirected binary networks that incorporated both spatial principles, the emerging networks revealed layouts that are stalwarts of the nervous system’s functional anatomy, such as parallelism, super-rings, and super-chains, while they maintained the complex network properties generated by adaptive rewiring [27].

### Convergent-divergent units

**Fig 2:**
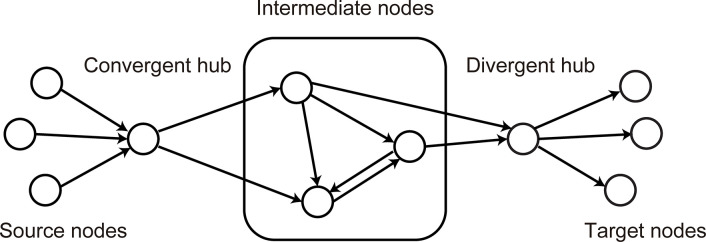
Schema of a convergent-divergent unit. In a convergent-divergent unit, a convergent hub collects inputs and passes the information to a divergent hub through a subnetwork of intermediate nodes. The nodes sending information to the convergent hub are referred as source nodes, and those receiving information from the divergent hub as target nodes. Note that typically the source and target nodes can show overlap, i.e., a node can be both a source and a target node.

Recent model developments have enabled extensions of the adaptive rewiring principle to undirected weighted [28,29] and directed binary graphs [30]. We study directed, weighted networks while incorporating both the spatial principles of distance minimization and alignment. Complementary to Calvo Tapia et al. [27] who were concerned with the network’s spatial layout, we focus on the evolution in these networks of a particular kind of structure that facilitates context-sensitive computation. In biological networks, context-sensitive computation is achieved through pooling, i.e., certain hub units collect converging inputs, and pass this information to divergent output-hubs via subnetworks of intermediate nodes (Fig 2). Such structures are known as convergent-divergent units [8,9]. Prominent examples of convergent-divergent units are the circuits in V1 underlying contextual modulation: pools of orientation selective neurons in layers 2/3 that send their input to somatostatin (SOM) cells, which then broadcast their response back to the network [31,32]. The SOM hub cells form with the vasoactive intestinal peptide (VIP) neurons an intermediate subunit between convergence and divergence that adjusts the contextual modulation response of the pools of neurons as the relationship between surround and stimulus changes [33]. At a different scale and scope, the cortico-basal ganglia circuitry can be seen as a convergent-divergent unit that regulates voluntary movement: the striatum receives multimodal contextual information from the cortex, processes it and sends it to other subcortical structures such as the pallidum and the substantia nigra, and then the thalamus, which acts as a divergent hub, broadcasting the processed output back to the cortex [34].

Convergent-divergent units thus constitute the connective core of sensory, motor, and cognitive brain regions (see [35] for a review), and of global networks [36]. They allow the receptive fields of sensory neurons to be driven by local features while being modulated by global contextual features [33]. This enables, among others, surround suppression via connections within area V1 [37,38] and sensorimotor prediction coding via long-range connections onto the visual system [39–41].

By employing adaptive rewiring to binary directed graphs, Rentzeperis et al. [30] observed in their model the emergence of convergent-divergent units. Here we use directed weighted graphs to study the effect of spatial optimization principles, i.e., distance minimization and topographic alignment, on the development of convergent-divergent units. We find that the distance minimization principle enables nodes to be encapsulated within convergent-divergent units. The alignment principle interferes with the formation of the convergent-divergent units, to an extent which depends on their layout.

Context-sensitivity differs across brain regions: more local for early and mid-level visual areas [42] and more global for higher order ones [43]. It also may vary between individuals [44], the sexes [45], and cultural groups [46]. These variations might be associated with variability in the convergent-divergent units. In our models, we find that the degree to which nodes within convergent-divergent units are encapsulated, or isolated from the rest of the network, depends parametrically on the prominence of distance minimization relative to adaptive rewiring, which enables tuning brain regions, or brain types, to their preferred processing style.

## Methods

### Notation and definitions

A directed graph (digraph) is described by the set, *G* = (*V*,*E*,*W*), where *V* = {1,2,…,*n*} is the set of nodes, *E*⊂*V*×*V* the set of ordered node pairs with (*j*,*i*)∈*E* representing directed edges from *j* to *i* denoted as *j*→*i*, and *W* ={*w*_*ij*_: *i*,*j*∈*V*} the set of edge weights, where *w*_*ij*_>0 if (*j*,*i*)∈*E*, and *w*_*ij*_ = 0 when (*j*,*i*)∉*E*. The cardinalities |*V*| = *n* and |*E*| = *m* denote the numbers of nodes and directed edges respectively.

**Fig 3:**
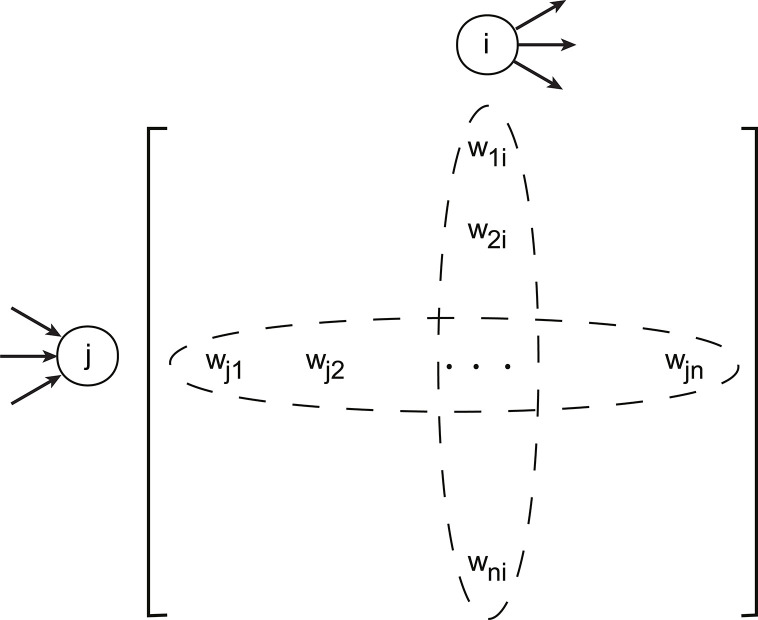
Schema of the adjacency matrix. The elements of the adjacency matrix are the weights of links. Each row of the adjacency matrix contains the weights of in-links for the corresponding node, and the number of nonzero entries is its in-degree. Similarly, each column carries the weights of out-links, and the number of nonzero entries is the out-degree.

Nodes are called adjacent if there is an edge (in either direction) between them. The *n*×*n* adjacency matrix *A* = [*A*_*ij*_]_*i*,*j*∈*V*_ carries the edge weights of a network as *A*_*ij*_ = *w*_*ij*_ (Fig 3). We refer to the edges directed at node *i*∈*V* as the in-link of *i* and the edges starting from node *i* as the out-link of *i*. In-strength and out-strength of a node quantify the strength of its incoming and outgoing connections, respectively. For a node *i*, its in-strength is defined as the sum of its in-link weights, $s_{i}^{in}=\sum_{j}A_{ij}$ while its out-strength the sum of its out-link weights, $s_{i}^{out}=\sum_{j}A_{ji}$.

The tails of the in-links of *i* constitute the in-degree neighborhood of *i*, *N*_*in*_(*i*). The remaining set of nodes, *V*−*i*−*N*_*in*_(*i*), is denoted as $N_{in}^{c}(i)$. The in-degree of node *i* is the number of its in-links. Analogously, the heads of the out-links of *i* constitute the out-degree neighborhood of *i*, *N*_*out*_(*i*), and the rest is denoted as $N_{out}^{c}(i)$. The out-degree of node *i* is the number of its out-links.

### Consensus and advection dynamics

Rentzeperis et al. [30] generalized the diffusion dynamics used for undirected graphs in Jarman et al. [20] to consensus and advection dynamics in digraphs. Both consensus [47] and advection dynamics [48] drive the network to converge to a global state based on the local state of each node. Each node’s local state is described by a value, referred to as concentration. In terms of neuronal dynamics, consensus and advection can be thought of as homeostatic mechanisms that reduce differences in activity between neurons. Because of their homeostatic property, consensus and advection do not require explicit modeling of inhibitory units to regulate the activity of the nodes. We consider our nodes as representing pools of excitatory neurons, on which the balancing dynamics of inhibitory neurons is implicitly modeled.

Consensus dynamics [47] is based on the diffusion Eq (1). The diffusion equation describes how a quantity diffuses in a medium across time,

$$\frac{\partial u}{\partial t}=\nabla \cdot (D\cdot \nabla u)$$ (1)

Where *u* is a scalar field representing the concentration of the quantity and *D* is the diffusion rate of the medium [49]. The discrete calculus analogue of the diffusion equation on an undirected network is

$${\dot{x}}_{i}(t)=\sum_{j\neq i}w_{ij}(x_{j}(t)-x_{i}(t))$$ (2)

where *x*_*i*_(*t*) is the concentration of node *i*, *i* =1,…,*n* [50]. The graph Laplacian matrix, *L*, is defined as *D*–*A*, where *D* = *diag*(*s*_1_,…,*s*_*n*_) (note that in the undirected case, the in-strength $s_{i}^{in}$ and out-strength $s_{i}^{out}$ of each node are equal since *A* is symmetric). The matrix form of the diffusion equation on an undirected network is

$$\dot{x}(t)=-Lx(t)$$ (3)

The solution is

$$x(t)=e^{-Lt}x(0)$$ (4)

where *x*(0) is the initial concentrations of the nodes.

Consensus dynamics naturally extends the diffusion Eqs (2) to (5), in which the direction of the links is taken the into account:

$${\dot{x}}_{i}(t)=\sum_{\left\{\forall j|j\to i\right\}}w_{ij}(x_{j}(t)-x_{i}(t))$$ (5)

The in-degree Laplacian matrix, *L*_*in*_, is defined as *D*_*in*_−*A*, where $D_{in}=diag(s_{1}^{in},\ldots ,s_{n}^{in})$. Then the consensus dynamics in matrix form becomes

$$\dot{x}(t)=-L_{in}x(t)$$ (6)

The concentration of the nodes at time t is

$$x(t)=e^{-L_{in}t}x(0)$$ (7)

Advection dynamics [48] is based on the advection Eq (8). The advection equation describes how a quantity is transported by a flow,

$$\frac{\partial u}{\partial t}=-\nabla \cdot (\vec{v}u)$$ (8)

where *u* is a scalar field representing the concentration of the quantity and $\vec{v}$ is the velocity vector of the flow [51]. For the directed network, the weight *w*_*ij*_ is identified as the flow velocity along link *j*→*i*, and the discrete calculus analogue of the advection equation reads

$${\dot{x}}_{i}(t)=\sum_{\left\{\forall j|j\to i\right\}}w_{ij}x_{j}(t)-\sum_{\left\{\forall k|i\to k\right\}}w_{ki}x_{i}(t)$$ (9)

where *x*_*i*_(*t*) is the concentration of node *i*, *i* =1,…,*n* [48]. The out-degree Laplacian matrix, *L*_*out*_, is defined as *D*_*out*_−*A*, where $D_{out}=diag(s_{1}^{out},\ldots ,s_{n}^{out})$. The advection dynamics in matrix form is:

$$\dot{x}(t)=-L_{out}x(t)$$ (10)

and the solution is:

$$x(t)=e^{-L_{out}t}x(0)$$ (11)

Note that the only difference between the solutions of consensus dynamics (7) and advection dynamics (11) consists in their respective Laplacian matrices. For the undirected case, where the adjacency matrix *A* is symmetric, *L*_*in*_ = *L*_*out*_ = *L*_,_ and both (7) and (11) equal to (4).

We refer to the exponential parts of (7) and (11) as the consensus kernel (12) and advection kernel (13), respectively.


$$c(t)=e^{-L_{in}t}$$ (12)

$$a(t)=e^{-L_{out}t}$$ (13)

The consensus and advection kernels are completely determined by the network structure. The (*i*,*j*) entry of the kernels characterizes the proportion of signal flow from node *j* to node *i* via all paths for a period of time (indicated by the variable *t*). Thus, the intensity of in-flow from other nodes to node *v* is proportional to the *v*th row vector of the kernel, and, analogously, the intensity of out-flow from *v* to other nodes is proportional to the *v*th column vector of the kernel. For the purposes of our study, the time variable *t* for the two kernels could also be thought of as a rewiring interval, the time elapsed between two successive rewiring steps. In all our experiments its value was set to 1.

### Rewiring principles

We probe how the structure of the network changes when we iteratively rewire its edges. In general, at each iteration, a node *v*∈*V* is randomly selected and either one of its in-link is cut and a new in-link for *v* is added, or one of its out-link is cut and a new out-link for *v* is added.

Suppose we rewire an in-link of *v* at iteration step *T*. The adjacency matrix before and after rewiring at this step are denoted by $A^{T-1}={\left[w_{ij}\right]}_{i,j\in V}^{T-1}$ and $A^{T}={\left[w_{ij}\right]}_{i,j\in V}^{T}$, respectively. During rewiring, an in-link of *v*, (*k*,*v*), will detach from *k* and reconnect to *l* which is not connected to *v* before rewiring, i.e., $w_{vk}^{T-1}>0$ and $w_{vl}^{T-1}=0$ becomes $w_{vk}^{T}=0$ and $w_{vl}^{T}=w_{vk}^{T-1}$. Thus, the number and strength of *v*’s in-links remain constant, but the number of out-links of other nodes could change (*k* has one less out-link, and *l* one more). Similarly, when an out-link of *v* is rewired, an out-link of *v*, (*v*,*k*), is substituted by a new out-link (*v*,*l*), i.e., $w_{kv}^{T-1}>0$ and $w_{lv}^{T-1}=0$ becomes $w_{kv}^{T}=0$ and $w_{lv}^{T}=w_{kv}^{T-1}$. In this case the number and strength of *v*’s out-links remain constant, but *k* has one in-link less, while *l* has one more.

To decide the choice of *k* and *l* at each rewiring step, one of the following three principles, explained below, will be selected with a fixed probability: either the functional principle of adaptive rewiring or one of the two spatial principles: the distance or the wave principle.

### Functional principle: Adaptive rewiring

The adaptive rewiring principle is called a functional principle, as it depends on the activation flow between nodes. It states that an underused connection is removed, and a new connection is established between two previously unconnected nodes with the most intense traffic between them (via all indirect paths). Distinct topological patterns develop when rewiring the in-degree neighborhood with the consensus algorithm and when rewiring the out-degree neighborhood with the advection algorithm [30]. Therefore, the *v*th row vector of the consensus kernel is used to represent the intensity of in-flow to *v* when we rewire the in-link of node *v*; the *v*th column vector of the advection kernel is used to represent the intensity of out-flow from *v* when we rewire the out-link of *v*.

When an in-link of *v* is rewired, *k* is the node in *N*_*in*_(*v*) such that link (*k*,*v*) has the lowest consensus kernel value, i.e., $k=argmin_{u\in N_{in}(v)}\{c{\left(t\right)}_{vu}\}$ ((*k*,*v*) is cut), and *l* is the node in $N_{in}^{c}(v)$ such that (*l*,*v*) has the largest consensus kernel value, i.e., $l=argmax_{u\in N_{in}^{c}(v)}\{c{\left(t\right)}_{vu}\}$ ((*l*,*v*) is added). In a similar fashion, when rewiring an out-link, *k* is the node in *N*_*out*_(*v*) such that (*v*,*k*) has the lowest advection kernel value, i.e., $k=argmin_{u\in N_{out}(v)}\{a{\left(t\right)}_{uv}\}$, and *l* is the node in $N_{out}^{c}(v)$ such that (*v*,*l*) has the largest advection kernel value, i.e., $l={argmax}_{u\in N_{out}^{c}(v)}\{a{\left(t\right)}_{uv}\}$. If maximums or minimums are tied, we randomly choose among the ties with uniform probabilities.

### Stochastic adaptive rewiring

Instead of deterministically selecting which connections to cut and which to add based on the maximum and minimum values of the consensus and advection kernels, we could use the kernels to assign probabilities and therefore add a stochastic component to the process. The stochastic adaptive rewiring is explained below.

When an in-link of *v* is rewired, we assign probabilities to the candidate links to be cut and to the candidate new links based on the consensus kernel values {*c*(*t*)_*vi*_, *i*≠*v*}. For the cutting of a connection, we choose node *k* from *N*_*in*_(*v*) with probability *p*_*cut*_(*k*):

$$p_{cut}(k)=\frac{\frac{1}{c{\left(t\right)}_{vk}}}{\sum_{i\in N_{in}(v)}\frac{1}{c{\left(t\right)}_{vi}}}$$ (14)

Thus in-links with lower kernel values have higher probability to be cut.

For the addition of a connection, node *l* is chosen from $N_{in}^{c}(v)$ with probability *p*_*add*_(*l*):

$$p_{add}(l)=\frac{c{\left(t\right)}_{vl}}{\sum_{i\in N_{in}^{c}(v)}c{\left(t\right)}_{vi}}$$ (15)

Unlike the probabilities for cutting a link, the probabilities for adding a link are analogous to the kernel values (normalized so that they sum to 1). If *c*(*t*)_*vi*_ = 0 for all $i\in N_{in}^{c}(v)$, we choose node *l* randomly from $N_{in}^{c}(v)$ with uniform probability.

Analogously, when rewiring an out-link, the probabilities for cutting and adding are:

$$p_{cut}(k)=\frac{\frac{1}{a{\left(t\right)}_{kv}}}{\sum_{i\in N_{out}(v)}\frac{1}{a{\left(t\right)}_{iv}}}$$ (16)

and

$$p_{add}(l)=\frac{a{\left(t\right)}_{lv}}{\sum_{i\in N_{out}^{c}(v)}a{\left(t\right)}_{iv}}$$ (17)

respectively.

### Spatial principles: Spatial rewiring

To instantiate the remaining two principles, the digraph is embedded in a two-dimensional Euclidean space, where the coordinates of node *i* are denoted as ${\vec{x}}_{i}\in R^{2}$.

### Distance principle

According to the distance principle, the longest connection is removed and replaced by the spatially closest connection possible between two previously unconnected nodes. The spatial distance between node *i* and node *j* is given by $d_{ij}=\|{\vec{x}}_{i}-{\vec{x}}_{j}\|$, where ‖∙‖ is the Euclidean distance. When an in-link of *v* is rewired, *k* is the node in *N*_*in*_(*v*) with the longest spatial distance from *v*, i.e., $k=argm{ax}_{u\in N_{in}(v)}\{d_{uv}\}$, and *l* the node in $N_{in}^{c}(v)$ with the shortest spatial distance from *v*, i.e., $l=argm{in}_{u\in N_{in}^{c}(v)}\{d_{uv}\}$. When rewiring an out-link of *v*, *k* is $argmax_{u\in N_{out}(v)}\{d_{vu}\}$ and *l* is $argmin_{u\in N_{out}^{c}(v)}\{d_{vu}\}$.

### Wave principle

The wave principle serves to optimize topographic alignment between network connections. It removes the connection at the largest angle to a vector field $\vec{F}(\vec{x})$ and replaces it with a connection between two previously unconnected nodes whose direction is most closely aligned with the direction of the vector field. The cosine of the angle between the edge *j*→*i* and the vector field at ${\vec{x}}_{v}$ is $cos(\theta_{ij})=\frac{\left({\vec{x}}_{i}-{\vec{x}}_{j})∙\vec{F}({\vec{x}}_{v}\right)}{d_{ij}\|\vec{F}({\vec{x}}_{v})\|},\theta_{ij}\in [0,\pi ]$. When rewiring an in-link of *v*, *k* is the node in *N*_*in*_(*v*) such that (*k*,*v*) forms the largest angle with $\vec{F}({\vec{x}}_{v})$, i.e., $k=argmin_{u\in N_{in}(v)}\{cos(\theta_{uv})\}$, and *l* is the node in $N_{in}^{c}(v)$ such that (*l*,*v*) forms the smallest angle with $\vec{F}({\vec{x}}_{v})$, i.e., $l=argmax_{u\in N_{in}^{c}(v)}\{cos\left(\theta_{uv}\right)\}$. When rewiring an out-link of *v*, *k* is $argmin_{u\in N_{out}(v)}\{cos(\theta_{vu})\}$ and *l* is ${argmax}_{u\in N_{out}^{c}(v)}\{cos(\theta_{vu})\}$.

### Rewiring algorithm

Throughout the rewiring process, the number of nodes and edges of the networks are kept constant for the sake of simplicity (but see [5] for growing and [52] for pruning in undirected networks). The rewiring process starts from a random directed network *D* = (*V*,*E*,*W*) with predetermined node number *n* and edge number *m*. Edges are assigned to *m* nodes pairs that are randomly selected from all *n*(*n*−1) node pairs without replacement. Then positive weights sampled from a normal distribution are randomly assigned to these edges. The probability to rewire an in-link at one iteration, *p*_*in*_, is set to a value between zero and one and the probability to rewire an out-link, *p*_*out*_, is its complementary, 1−*p*_*in*_. The probabilities of choosing the distance principle, *p*_*distance*_, wave principle, *p*_*wave*_, and functional principle *p*_*function*_ are also predetermined and sum to one: *p*_*function*_ = 1−*p*_*distance*_−*p*_*wave*_.

The iterative rewiring process proceeds as follows.

Step 1: Generate a random number *r*_1_ from a uniform distribution *U*[0,1]. If *r*_1_<*p*_*in*_, select a random node *v*∈*V* such that its in-degree is neither zero nor *n*−1. Otherwise, select a random node *v*∈*V* such that its out-degree is neither zero nor *n*−1

Step 2: Generate a random number *r*_2_ from a uniform distribution *U*[0,1]. If *r*_2_<*p*_*distance*_, the distance principle is chosen; if *p*_*distance*_≤*r*_2_<*p*_*distance*_+*p*_*wave*_, the wave principle is chosen; if *p*_*distance*_+*p*_*wave*_≤*r*_2_, the functional principle is chosen.

Step 3: If *r*_1_<*p*_*in*_, rewire the in-link of *v*, otherwise rewire the out-link of *v*, in both cases according to the principle chosen in Step 2.

Step 4: Return to step 1 until *M* edges have been rewired.

We refer to this algorithm as the ‘functional + spatial’ algorithm.

### Baseline algorithm

We take the algorithm from Rentzeperis et al. [30] as the baseline algorithm, in which the functional principle was combined with random rewiring and no spatial principles were used. Random rewiring drops and adds links randomly. Suppose that an in-link of a node *v*∈*V* is rewired. According to random rewiring, two nodes *k*∈*N*_*in*_(*v*) and $l\in N_{in}^{c}(v)$ are selected randomly. The in-link (*k*,*v*) is cut and (*l*,*v*) is added. Random rewiring for out-links is similar. Note that random rewiring uses uniform probabilities for selecting nodes *k* and *l*, but stochastic adaptive rewiring uses probabilities that depend on the kernel values of consensus or advection. The probabilities of choosing functional principle and random rewiring at each iteration are *p*_*function*_ and *p*_*random*_ (*p*_*random*_ = 1−*p*_*function*_), respectively. This algorithm is referred as the ‘functional + random’ algorithm.

This algorithm was originally applied to directed binary networks. We run the ‘functional + random’ algorithm on directed weighted networks to test if similar results are obtained and compare its effects to those of the ‘functional + spatial’ algorithm.

### Network measures

To study the impact of the rewiring principles on the structure of weighted digraphs, we calculate the following measures for each rewired network. High scores on each of these measures reflect better information processing and communication within the network.

### Average efficiency

The average efficiency metric quantifies the efficiency of sending information over a network, which is defined as the mean of the inversed shortest directed path lengths of all node pairs [53]. Whereas the networks are spatially embedded, we are interested in the efficiency of the network in terms of its connectivity pattern and weights. Thus, we define a topological distance, $l_{ij}=\frac{1}{w_{ij}}$, which can be interpreted as the difficulty of transmission [54]. At the neuronal level, the stronger the synapse (large *w*_*ij*_) is, the easier electric nerve impulses can transmit between two neurons (small ℓ_*ij*_).

For an ordered node pair (*u*,*v*), a directed walk from *u* to *v* is an ordered list of edges $\left\{(i_{0},i_{1}),(i_{1},i_{2}),\ldots ,(i_{K-1},i_{K}):i_{0}=u,i_{K}=v,(i_{k-1},i_{k})\in E\right\}$ [55]. A directed walk is a directed path if the vertices on it are distinct. Average efficiency is then defined as:

$$E=\frac{1}{n(n-1)}\sum_{i\neq j\in V}\frac{1}{l_{ij}}$$ (18)

where ℓ_*ij*_ is the length of the shortest directed path from node *i* to node *j*, i.e., the easiest transmission route from node *i* to node *j*. If there is no transmission route from *i* to *j*, ℓ_*ij*_ = ∞.

### Number of connected node pairs

An ordered pair (*i*,*j*) is connected if there is a directed path from *i* to *j*. The number of connected node pairs is a measure of the extent of information exchange in a digraph. The upper bound of the number of connected node pairs is *n*^2^ which is achieved when every node can send information to any node, including itself. We use this measure to quantify the connectedness of a digraph.

### Number of hubs

We define convergent hubs as nodes with at least one out-link and a number of in-links that are above a threshold we define. These hubs are suitable as a substrate for collecting distributed information. Inversely, divergent hubs are nodes with at least one in-link and a number of out-links above a predefined threshold. These hubs are suitable as a substrate for information broadcasting. The threshold was set to 15 for both convergent and divergent hubs in the following analysis.

### Simulation parameter settings

In our simulations, the number of nodes was *n* = 100. We set the number of edges to m=[2(n)*(n−1)]=912, a number sufficiently low for a network to be considered sparse (cf. [52] for undirected networks). The unnormalized weights were sampled from a normal distribution: *N*(1, 0.25^2^). Sampled negative weights from the normal distribution (an almost impossible occurrence as indicated by its probability: 3.17*10^−5^) were set to 0.05. Normalized weights were obtained by dividing the sampled weights by the sum of all weights. That way the sum of the new normalized weights equals to the number of edges.

For the purposes of spatial embedding, nodes were placed randomly with a uniform distribution on a unit disk; the external field was set to a field $\vec{F}(\vec{x})=(1,0)$ to induce parallel connections or a radial field $\vec{F}(\vec{x})=\frac{\vec{x}}{\left\|\vec{x}\right\|}$ to induce concentric connections.

The number of steps *M* for each run was 15000. The probabilities of the three principles (*p*_*function*_, *p*_*distance*_, *p*_*wave*_) and the probability of rewiring in-link *p*_*in*_ were kept fixed for each run. For each combination of parameters (*p*_*in*_, *p*_*function*_, *p*_*distance*_, *p*_*wave*_), we run 10 different instantiations of the rewiring algorithm, over which the mean and standard deviation of the measures were calculated after 15000 rewiring steps.

## Results

We first examine how each rewiring principle drives the evolution of the network’s spatial layout and topology. Then we probe for the optimal combination of adaptive and random rewiring for producing convergent-divergent units. Subsequently, we test whether the distance principle facilitates in the formation of convergent-divergent units similarly to random rewiring. We then probe whether the wave principle has any effect in the production of convergent-divergent units. Finally, we examine whether stochastic adaptive rewiring further facilitates the generation of convergent-divergent units. We found similar results when the weights of the connections followed a lognormal distribution (not shown here).

### Evolution of networks

We offer some examples illustrating the effects of each rewiring principle on the evolution of networks. Starting from the same initial random network, we generate one instance for each rewiring principle.

**Fig 4:**
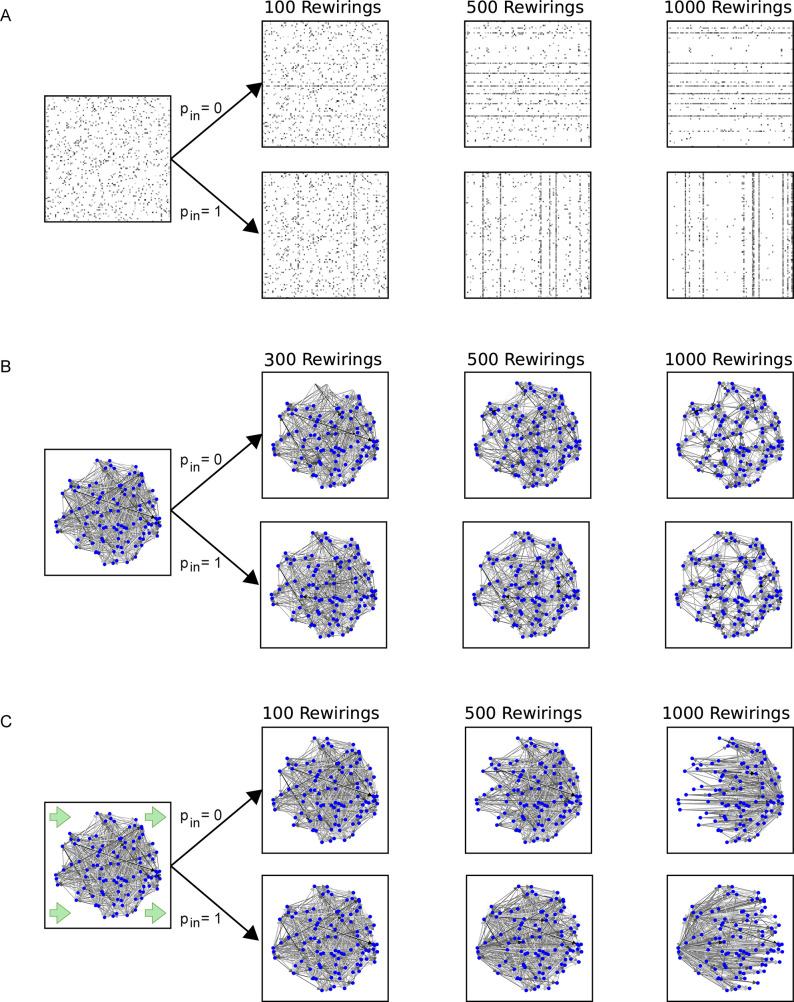
Rewiring based on the functional principle develops winner-take-all configurations, based on the distance principle forms clusters, and based on the wave principle aligns the connections with the latent field. (A) Evolution of the adjacency matrix driven by the functional principle only. (B) Evolution of the network spatial layout driven by the distance principle only. (C) Evolution of the network spatial layout driven by the wave principle only when the wave propagates laterally. In all cases, we either rewire the out-links (*p*_*in*_ = 0 case) or the in-links (*p*_*in*_ = 1 case). Link weights follow the normal distribution.

When we apply only functional rewiring (*p*_*cunction*_ = 1), networks change their topology swiftly. They start to develop winner-take-all configurations just after 100 rewiring steps, in that certain nodes receive input from many other nodes (the rows of the adjacency matrix, *p*_*in*_ = 0 in Fig 4A; convergent part) and other nodes broadcast their output to many other nodes (the columns of the adjacency matrix, *p*_*in*_ = 1 in Fig 4A; divergent part). The value of *p*_*in*_ controls the proportion of convergent or divergent hubs formed. The functional principle does not have a visible impact on the spatial layout of the digraphs (S1 Fig).

When we apply only the distance principle (*p*_*distance*_ = 1), the adjacency matrices appear random (S2 Fig), but their spatial representations show cluster formation after 500 steps (Fig 4B).

When we apply only the wave principle (*p*_*wave*_ = 1), the spatial layout of the network shows alignment to the propagating wave after 300 steps (Figs 4C and S3). Observation of some of the columns or rows of the adjacency matrices suggests the initiation of hub formation (S4 Fig). This effect, however, is much weaker than the one derived from functional rewiring.

### Formation of convergent-divergent units on directed weighted networks

**Fig 5:**
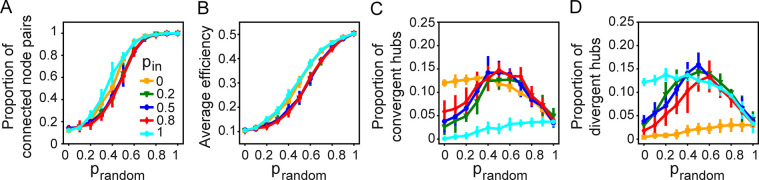
Random rewiring enhances connectedness and increases the number of hubs when rewiring includes both advection and consensus (0<*p*_*in*_<1). (A) The proportion of connected node pairs, (B) average efficiency, (C) proportion of convergent hubs, and (D) proportion of divergent hubs as a function of *p*_*random*_, for different *p*_*in*_.

We run the ‘functional + random’ algorithm on directed weighted networks for various combinations of *p*_*function*_ and *p*_*random*_ values. Τhe emergence of convergent-divergent units requires the formation of both convergent and divergent hubs as well as the existence of communication pathways between them. In general, the number of node pairs that are connected via a directed path increases with the proportion of random rewiring, *p*_*random*_, regardless of the proportion of in-link rewiring, *p*_*in*_ (Fig 5A). The effect of *p*_*random*_ on efficiency (Fig 5B) is similar with that on connectedness (Fig 5A), which implies that the increase of the networks’ efficiency can be attributed mostly to their increase of connectedness. As expected, the number of convergent hubs decreases while the number of divergent hubs increases when *p*_*in*_ increases (Fig 5C and 5D) and for 0<*p*_*in*_<1, the number of convergent and divergent hubs peaks at intermediate *p*_*random*_ values (Fig 5C and 5D).

We subsequently asked whether the network achieves steady-state, and if so after how many rewiring steps. We found that connectedness as well as average efficiency reach a relatively stable value after about 2500 rewiring steps while the proportion of convergent and divergent hubs need more rewiring steps to reach a relatively stable value (S5 Fig). This, however, does not mean that the network is static. Hubs can lose their connections and other nodes with initially few connections can become hubs. This indicates that the network continues to show plasticity throughout its development, a property that may be beneficial for learning.

**Fig 6:**
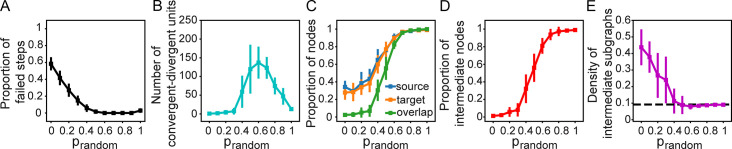
*p*_*random*_ controls the formation, connectedness, and degree of isolation of convergent-divergent units. (A) Proportion of steps with no convergent-divergent unit in the network, (B) number of convergent-divergent units in rewired networks, (C) proportion of source nodes, target nodes and their overlap, (D) proportion of nodes in intermediate subgraphs and (F) density of intermediate subgraphs as a function of *p*_*random*_. The black horizontal line is the density of the whole digraph.

We examined whether the convergent and divergent hubs in the network are connected in a way that they can form convergent-divergent units, i.e., they have at least a convergent hub, a divergent hub, and a communication pathway (a directed path) between them. The probability of rewiring in-links, *p*_*in*_, is set to 0.5, so that equal proportions of convergent and divergent hubs could develop. We found that convergent-divergent units emerge when adaptive and random rewiring are combined (Fig 6A and 6B). The proportion of random rewiring, *p*_*random*_, controls the stability of the formation of convergent-divergent units (Fig 6A). When *p*_*random*_ is low, convergent-divergent units frequently disappear during the rewiring process because of the lack of communication pathways. The number of convergent-divergent units initially increases with increasing *p*_*random*_, but drops for *p*_*random*_>0.6 (Fig 6B). At low *p*_*random*_ values, convergent and divergent hubs exist (Fig 5C and 5D), but they oftentimes do not connect to each other (Fig 5A), so that the number of convergent-divergent units is close to or exactly 0 (Fig 6B). On the other hand, for large *p*_*random*_ values, the number of convergent-divergent units decreases (Fig 6B) because there are fewer convergent and divergent hubs in networks (Fig 5C and 5D).

The connectedness of the hub nodes also depends on the proportion of random rewiring. For a convergent-divergent unit, we refer to nodes that can be reached from the divergent hub as the target nodes and the nodes that send information to the convergent hub as the source nodes (Fig 2). The proportions of source and target nodes as well as their proportion of overlap (a source node also being a target node) increase with *p*_*random*_ (Fig 6C).

For each convergent-divergent unit, we refer to nodes on directed paths from the convergent to the divergent node as intermediate nodes, and to the subgraph which consists of these nodes as the intermediate subgraph. The intermediate subgraph processes the information collected by the convergent hub. The degree of its isolation from the rest of the network characterizes the context-sensitivity of its processing style. We calculated for all convergent-divergent node units, the size and density of the intermediate subgraph, as long as this subgraph contained more than one node. For each combination of (*p*_*function*_, *p*_*random*_), the sizes and densities of the subgraphs were pooled together across 10 instances.

We found that the size of the subgraph increases with *p*_*random*_ (Fig 6D) while the average density decreases until it reaches and stays at a floor value at *p*_*random*_>0.5, near the density of the whole digraph (Fig 6E) because all of the nodes of the graph except for the convergent and divergent hubs become part of it.

### Distance-based rewiring has effects on the network similar to random rewiring

We replaced random rewiring with distance-based rewiring to see the latter’s effect on the network. We run the ‘functional + spatial’ algorithm without including wave-based (*p*_*wave*_ = 0) or random rewiring.

**Fig 7:**
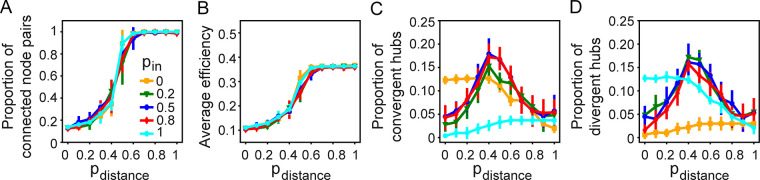
Distance-based rewiring has similar effects on the connectedness and the number of hubs as random rewiring. (A) Proportion of connected node pairs, (B) average efficiency, (C) proportion of convergent hubs, and (D) proportion of divergent hubs as a function of *p*_*distance*_, for different probabilities of in-link rewiring, *p*_*in*_.

We found that distance-based rewiring, *p*_*distance*_, has an effect similar to random rewiring on connectedness, average efficiency, and hub formation (compare Fig 7 with Fig 5). Note however that *p*_*random*_ yields higher average efficiency compared to *p*_*distance*_ (compare Fig 7B with Fig 5B). Adding a small proportion of random rewiring into the combination of adaptive rewiring and distance-based rewiring can increase average efficiency when the proportion of adaptive rewiring is low (S6 Fig).

**Fig 8:**
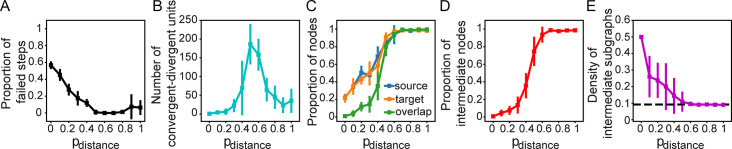
*p*_*distance*_, controls the formation, connectedness, and degree of isolation of convergent-divergent units. (A) Proportion of steps with no convergent-divergent unit in the network, (B) number of convergent-divergent units in rewired networks, (C) proportion of source nodes, target nodes and their overlap, (D) proportion of nodes in intermediate subgraphs and (E) density of intermediate subgraphs as a function of *p*_*distance*_. The black horizontal line represents the density of the whole digraph.

The failure rate of the formation of convergent-divergent units slightly increases with spatial rewiring compared to when using random rewiring (Fig 8A). The number of convergent-divergent units, connectedness of hubs, and the size and density of intermediate subgraphs vary with *p*_*distance*_ in a similar fashion as with *p*_*random*_ (Fig 8B–8E). The average density decreases until *p*_*distance*_≥0.5, then remains stable (Fig 8E). As long as *p*_*distance*_<0.5, we can regard the intermediate nodes as encapsulated to various degrees from the rest of the network, regulated by *p*_*distance*_. This effect is independent of the modularity of the network structure, which changes proportionally with *p*_*distance*_ (S7 Fig). These results indicate that random rewiring can effectively be replaced by spatially-based rewiring according to the distance principle.

### Variable effects of the wave principle on convergent-divergent units depending on the field

**Fig 9:**
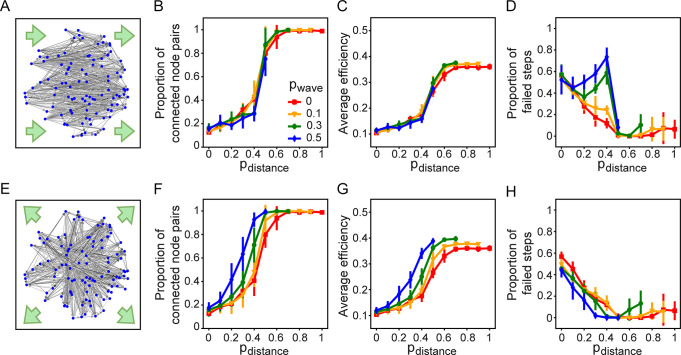
The way the wave principle affects the formation of convergent-divergent units depends on the underlying field. (A) Spatial layout of a network evolved with a lateral field and (E) with a radial field. Green arrows indicate the direction of the underlying field. The proportion of in-link rewiring is 0.5, and (*p*_*function*_, *p*_*distance*_, *p*_*wave*_) is (0.4,0.3,0.3). (B-D) The proportion of connected node pairs, average efficiency, and the proportion of steps with no convergent-divergent unit in the network, as a function of the distance-based principle, *p*_*distance*_, with a lateral field, and (F-H) with a radial field.

When included, wave-based rewiring affects the network’s spatial layout (Fig 9A and 9E), but it does not modulate the effect of distance-based rewiring on hub formation (S8A and S8B Fig). We found, however, that the way wave-based rewiring affects the network depends on the field. For the lateral field, when *p*_*wave*_>0.1 and 0.3≤*p*_*distance*_≤0.4, wave-based rewiring dampens the connectedness, and has little impact on average efficiency (Fig 9B and 9C). For the radial field, it promotes the connectedness and average efficiency (Fig 9F and 9G). The formation of convergent-divergent units also depends on the underlying field (Fig 9D and 9H). For the lateral field, wave-based rewiring typically has a detrimental effect (Fig 9D), while for the radial field it can marginally improve the formation of convergent-divergent units for *p*_*distance*_≤0.4 (Fig 9H). Other metrics of convergent-divergent units are also dependent on the underlying field (S9–11 Figs).

### Stochastic adaptive rewiring improves the formation of convergent-divergent units

**Fig 10:**
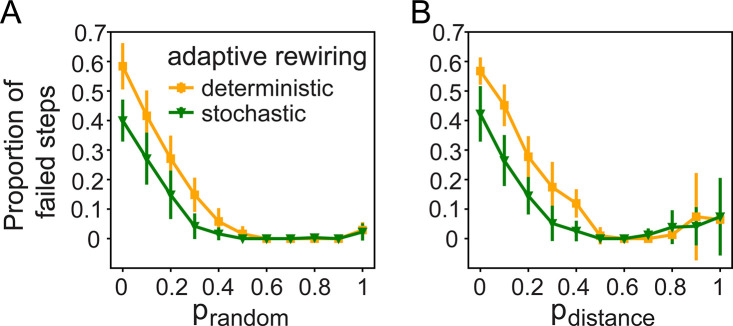
Stochastic adaptive rewiring reduces the number of steps with no convergent-divergent unit is in the network. The proportion of in-link rewiring, *p*_*in*_, is 0.5. The proportion of steps with no convergent-divergent unit in the network, as a function of (A) *p*_*random*_, and (B) *p*_*distance*_.

Our results show that random rewiring improves a network’s connectedness and efficiency and facilitates its development of convergent-divergent units. We asked if we would see further improvements on the networks if we replaced the standard deterministic adaptive rewiring with a stochastic version that assigns probabilities to the kernels instead of always picking the minimum kernel value for cutting and the maximum for adding connections. We combined stochastic adaptive rewiring with random or distance-based rewiring and set *p*_*in*_ to 0.5. Stochastic adaptive rewiring shows similar trends as the standard deterministic version (S12–14 Figs), but it improves the robustness of the convergent-divergent units (Fig 10) showing that this controlled stochasticity of rewiring further benefits the network.

### Discussion

Starting from a random graph, repeated application of adaptive rewiring leads to complex, brain-like network structures. Previous studies explored this phenomenon for directed binary and undirected graphs; here we extended the scope of this principle to directed weighted graphs and considered their spatial embedding [27]. Similar to these studies, at each rewiring step, we randomly chose with different proportions from three basic rewiring principles: the functional principle of adaptive rewiring according to its ongoing network activity, as represented here by generalized diffusion (i.e., advection and consensus dynamics [30]) and two spatial principles: wiring distance minimization and vector field alignment. All three principles are deemed important in shaping the layout of the nervous system [27].

We found that functional and spatial principles took complementary roles: whereas the adaptive rewiring principle takes the role of forming hubs, the distance minimization principle ruled over network connectedness and efficiency. Previous studies in adaptive rewiring without spatial principles found that adaptive rewiring only, while effective in forming hubs and modules, tends to reduce connectivity and efficiency [30]. In their seminal study, Watts & Strogatz [56] showed that adding a small proportion of random connections strongly improved the efficiency and connectedness of a modular network. For this reason, all previous adaptive rewiring studies, from Gong & van Leeuwen [5] to Rentzeperis et al. [30], introduced a proportion of random rewiring to their network models, thus securing efficiency and connectedness.

Replacing the completely deterministic adaptive rewiring rule, which always selects the minimum kernel value for cutting and the maximum kernel value for adding connections, with a stochastic version that assigns probabilities to the kernels, further improves the performance of the rewiring algorithm. Stochastic adaptive rewiring yields more robust convergent-divergent units. The result suggests that this sort of controlled stochasticity may provide better stability in the maintenance of structures in the brain than the hitherto preferred addition of completely random rewiring.

As spatial rewiring principles are shown to play a similar role to random rewiring they successfully substitute random rewiring in our model. A major difference between random and spatial rewiring is that the former benefits global connectivity [56], whereas the latter favors local connectivity [21]. This discrepancy, however, was shown to be no obstacle to the formation of hubs in the network. In fact, applying the distance principle showed the network to evolve a modular structure.

A limitation of the current study is that in our models, the relative contribution of all three rewiring principles was fixed during the network evolution. We did not consider the possibility that different rewiring principles change in prominence over time. Early in the development of the brain, topographical alignment may play a rather prominent role, as brain activity around gestation shows massive bursts of action potentials that spread in a wave-like manner [57]. By contrast, the formation of hubs continues over a much longer period that extends into late adolescence [23]. A further shortcoming, as noted, is that the balancing dynamics of excitatory and inhibitory neurons in the brain, and their role in the formation of brain structures, are modeled implicitly, based on the homeostatic properties of the advection/consensus dynamics. Although computationally convenient, a more realistic modeling approach may still be desirable. In future work, we aim to return to modeling adaptive rewiring in spiking neurons [17], explicitly incorporating the balancing dynamics of inhibitory neuron populations [58] to study their role in the development of brain structure.

In directed networks, we may distinguish convergent hubs and divergent hubs. Which of these is more prominent depends on the proportion of advection and consensus dynamics applied in the model [30]. This feature may be useful to customize networks to processing requirements, e.g., divergence may be more useful in early processing regions; convergence in later ones [59]. When the advection and consensus dynamics are balanced, adaptive rewiring forms equal numbers of convergent and divergent hubs. These are the major constituents of convergent-divergent units. Convergent-divergent units collect information from pools of nodes through the convergent hubs, process the information in intermediate nodes, and broadcast the results to the network through the divergent hubs. In the brain these units enable context-sensitive modulation of network activity. In the model, convergent-divergent units are formed when convergent and divergent hubs arise (due to adaptive rewiring) and, when the network is efficiently connected (due to the distance minimization principle). We found that, as long as adaptive rewiring and the distance minimization principle are balanced in the evolving network, convergent-divergent hubs are successfully formed.

The distance minimization principle thus interacted constructively with adaptive rewiring in the formation of convergent-divergent units. Moreover, it contributed modularity to the network and established a rich club effect amongst the hubs. Because of this, the units jointly constitute the connective core [36] of the network.

An important feature of the distance minimization principle is that its prominence in rewiring determines the degree of encapsulation of the intermediate nodes in the convergent units. With lower proportions of distance-based rewiring, the intermediate nodes were relatively isolated from the rest of the network; with higher proportions they were more interconnected with it. In other words, the relative contribution of the distance principle regulates the context-sensitivity of the computations performed in the convergent-divergent units. We may consider the possibility that this feature is used to establish that whole networks differ regarding the context-sensitivity of their processing style [44–46] or to tailor the convergent-divergent units of different subnetworks to their specific computational requirements [35,42,43].

Different types of convergent-divergent units could be assigned to different subnetworks. Several different subnetworks have been distinguished in the brain, such as the dorsal attention, [60,61], the salience [62,63] and the default mode network [64,65] that could operate in competition or in cooperation. The set of globally interconnected convergent-divergent units may constitute the global workspace [66,67]. Such an account would adequately differentiate the global workspace from the various functionally specialized networks, enabling a full dissociation of consciousness and attention [68,69].

In general, our rewiring algorithm could serve as a substrate for building null models for linking computational models with experimental data [70]. For instance, one could generate random networks with the same size and density as an empirical network and rewire them with either spatial principles or with a combination of spatial and functional principles. The two cases of rewired networks could give two null models and by comparing the empirical feature values to the ones from the null models, one may determine the roles of functional and spatial principles in the formation of the empirical network.

## Supporting information

S1 Fig Applying the functional principle of adaptive rewiring has no discernable impact on the spatial layout of the network. Evolution of network spatial layout when applying the functional principle only while exclusively rewiring either the out-links (*p*_*in*_ = 0) or the in-links (*p*_*in*_ = 1).
(DOCX)

S2 Fig Applying the distance principle has no discernable impact on the adjacency matrix of the network. Evolution of adjacency matrices when applying the distance principle only while exclusively rewiring either the out-links (*p*_*in*_ = 0) or the in-links (*p*_*in*_ = 1).
(DOCX)

S3 Fig With a wave propagating radially, the wave principle drives the network connections to alignment with the direction of the wave field. Evolution of network spatial layout when applying the wave principle while rewiring either the out-links (*p*_*in*_ = 0) or the in-links (*p*_*in*_ = 1).
(DOCX)

S4 Fig Applying the wave principle to the network starts forming hubs after 500 rewiring steps. Evolution of network connectivity when applying the wave principle and either rewire the (*p*_*in*_ = 0 case) or the in-links (*p*_*in*_ = 1 case). The wave propagates either (A) laterally or (B) radially.
(DOCX)

S5 Fig The proportion of connected node pairs, average efficiency, proportion of convergent and divergent hubs stabilize their values but never become completely static. (A) The ‘functional + random algorithm and (B) the ‘functional + spatial’ algorithm without wave-based rewiring (*p*_*wave*_ = 0), *p*_*in*_ = 0.5 for both.
(DOCX)

S6 Fig Including a small proportion of random rewiring into the combination of adaptive and distance-based rewiring can further increase average efficiency at low proportions of adaptive rewiring. Average efficiency as a function of the proportion of adaptive rewiring, *p*_*function*_, *p*_*in*_, was set to 0.5.
(DOCX)

S7 Fig Modularity is proportional to the probability of distance-based rewiring, *p*_*distance*_. Modularity as a function of *p*_*distance*_, for different probabilities of in-link rewiring, *p*_*in*_.
(DOCX)

S8 Fig Rewiring based on the wave principle does not change the emergence of convergent and divergent hubs as a function of the proportion distance-based rewiring. Proportion of convergent and divergent hubs as a function of *p*_*distance*_ (A) for the lateral and (B) the radial field.
(DOCX)

S9 Fig Wave-based rewiring reduces the number of convergent-divergent units in case of a lateral field, while increases it in case of a radial field. The number of convergent-divergent units in rewired networks as a function of *p*_*distance*_ for the lateral and the radial field.
(DOCX)

S10 Fig Wave-based rewiring dampens the proportion of source and target nodes and their overlap when *p*_*wave*_>0.1 and *p*_*distance*_ = 0.4 for the lateral field; it increases them when *p*_*wave*_>0.1 for the radial field. Proportions of source and target nodes and their overlap with *p*_*distance*_, for (A) the lateral and (B) radial field case. The dashed lines are for values when no wave-based rewiring is performed, i.e., *p*_*wave*_ = 0.
(DOCX)

S11 Fig Wave-based rewiring reduces the size of intermediate subgraphs at *p*_*distance*_ = 0.4 and *p*_*wave*_>0.1, but increases the density of intermediate subgraphs when *p*_*distance*_>0 for the lateral field; it increases the size of intermediate subgraphs when *p*_*distance*_>0.1, but does not change the density of intermediate subgraphs in a systematic way for the radial field case. (A) Proportion of nodes in intermediate subgraphs and (B) density of intermediate subgraphs as a function of *p*_*distance*_ for the lateral and the radial field.
(DOCX)

S12 Fig Using stochastic instead of the standard (deterministic) adaptive rewiring does not change the way *p*_*random*_ and *p*_*distance*_ control the proportion of connected node pairs, average efficiency, and the proportion of convergent and divergent hubs. (A) The ‘functional + random’ algorithm and (B) the ‘functional + spatial’ algorithm without wave-based rewiring (*p*_*wave*_ = 0), both for *p*_*in*_ = 0.5. Dashed lines correspond to the results of the standard (deterministic) adaptive rewiring, and solid lines to stochastic adaptive rewiring.
(DOCX)

S13 Fig The proportion of connected node pairs, average efficiency, convergent and divergent hubs stabilize but are never completely static. (A) The combination of stochastic adaptive rewiring and random rewiring. (B) The combination of stochastic adaptive rewiring and distance-based rewiring. *p*_*in*_ is 0.5 for both cases.
(DOCX)

S14 Fig Using stochastic adaptive rewiring instead of deterministic increases the number of convergent-divergent units. (A) Combination of stochastic adaptive rewiring and random rewiring, and (B) combination of stochastic adaptive rewiring and distance-based rewiring, both for *p*_*in*_ = 0.5. Dashed lines correspond to the results of the standard (deterministic) adaptive rewiring, solid lines to the stochastic adaptive rewiring.
(DOCX)
