# Supplementary material for: Functional and spatial rewiring principles jointly regulate context-sensitive computation
Source: PLoS Comput Biol. 2023 Aug 11;19(8):e1011325. doi: 10.1371/journal.pcbi.1011325 (PMC10446201; doi:10.1371/journal.pcbi.1011325)
Supplement: S1 Fig — Evolution of network spatial layout when applying the functional principle only while exclusively rewiring either the out-links (pin = 0) or the in-links (pin = 1). (DOCX) [file pcbi.1011325.s001.docx]

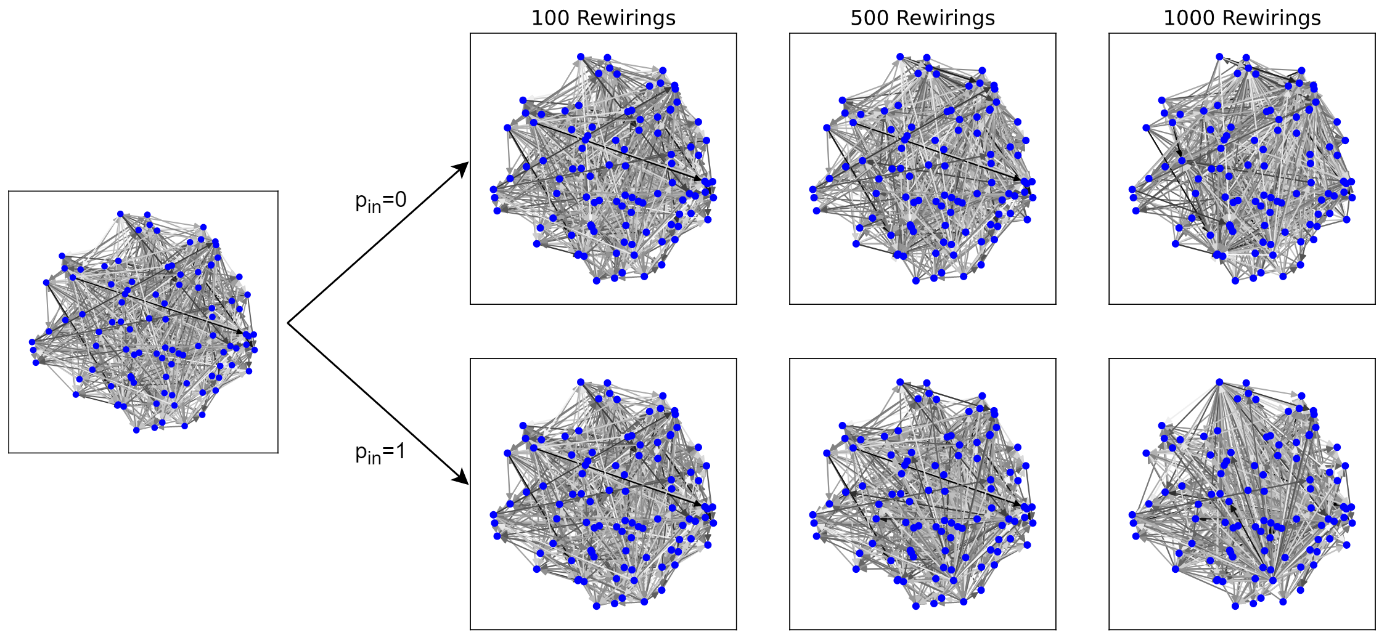


**Fig S1.** Applying the functional principle of adaptive rewiring has no discernable impact on the spatial layout of the network. Evolution of network spatial layout when applying the functional principle only while exclusively rewiring either the out-links (*p_in_* = 0) or the in-links (*p_in_* = 1).
