# Supplementary material for: Functional and spatial rewiring principles jointly regulate context-sensitive computation
Source: PLoS Comput Biol. 2023 Aug 11;19(8):e1011325. doi: 10.1371/journal.pcbi.1011325 (PMC10446201; doi:10.1371/journal.pcbi.1011325)
Supplement: S2 Fig — Evolution of adjacency matrices when applying the distance principle only while exclusively rewiring either the out-links (pin = 0) or the in-links (pin = 1). (DOCX) [file pcbi.1011325.s002.docx]

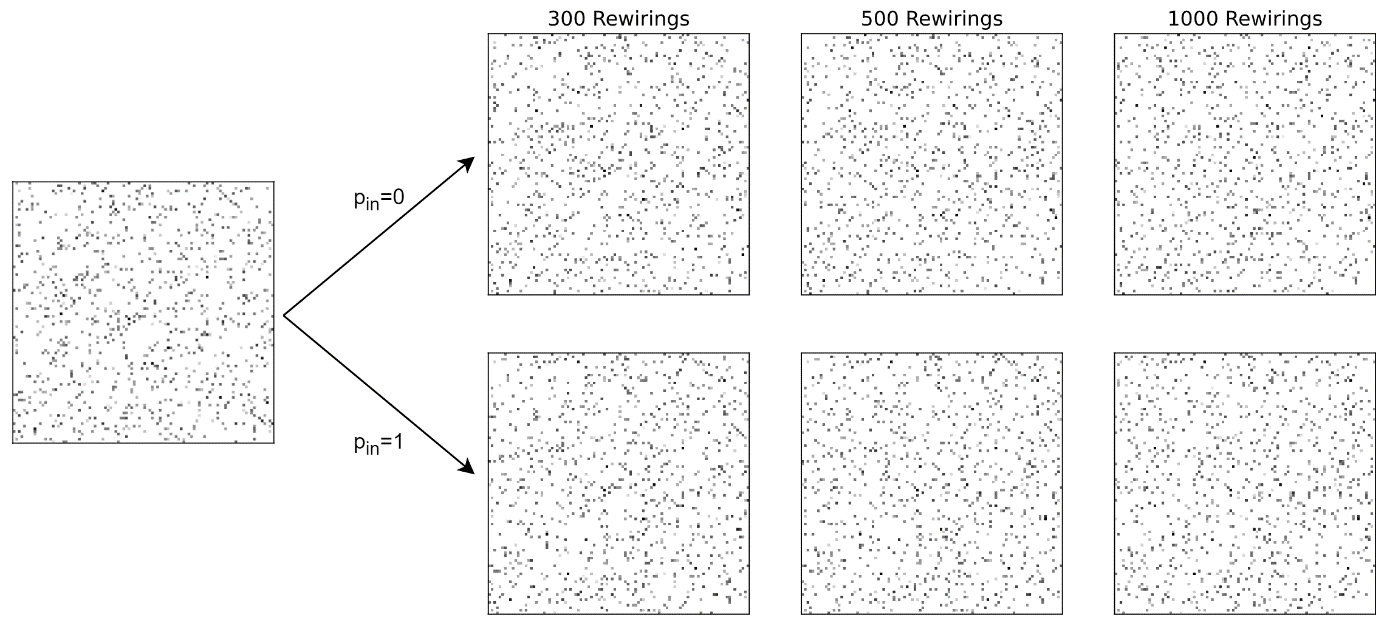


**Fig S2.** Applying the distance principle has no discernable impact on the adjacency matrix of the network. Evolution of adjacency matrices when applying the distance principle only while exclusively rewiring either the out-links (*p_in_* = 0) or the in-links (*p_in_* = 1).
