# Supplementary material for: Functional and spatial rewiring principles jointly regulate context-sensitive computation
Source: PLoS Comput Biol. 2023 Aug 11;19(8):e1011325. doi: 10.1371/journal.pcbi.1011325 (PMC10446201; doi:10.1371/journal.pcbi.1011325)
Supplement: S3 Fig — Evolution of network spatial layout when applying the wave principle while rewiring either the out-links (pin = 0) or the in-links (pin = 1). (DOCX) [file pcbi.1011325.s003.docx]

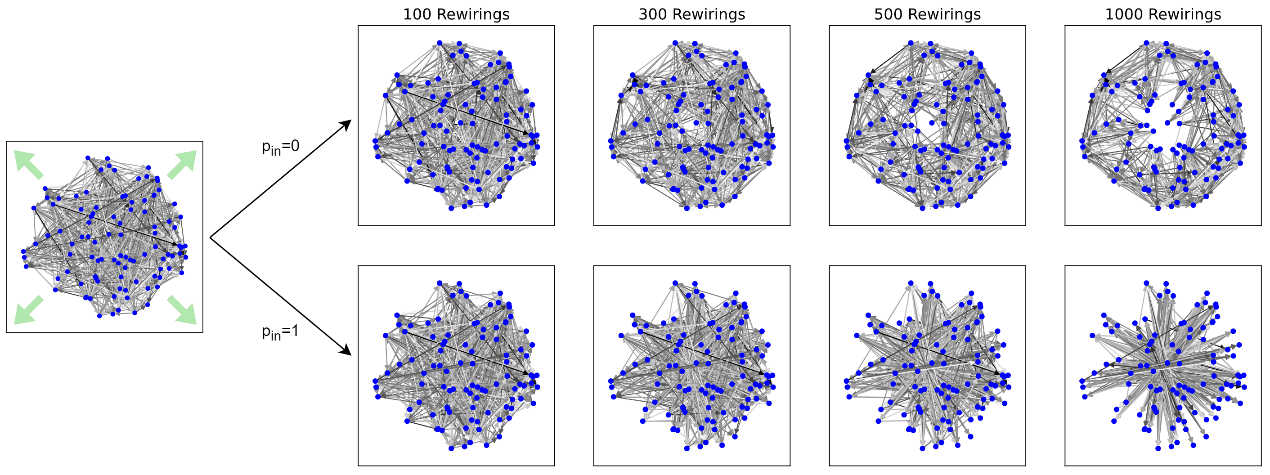


**Fig S3.** With a wave propagating radially, the wave principle drives the network connections to alignment with the direction of the wave field. Evolution of network spatial layout when applying the wave principle while rewiring either the out-links (*p_in_* = 0) or the in-links (*p_in_* = 1).
