# Supplementary material for: Functional and spatial rewiring principles jointly regulate context-sensitive computation
Source: PLoS Comput Biol. 2023 Aug 11;19(8):e1011325. doi: 10.1371/journal.pcbi.1011325 (PMC10446201; doi:10.1371/journal.pcbi.1011325)
Supplement: S4 Fig — Evolution of network connectivity when applying the wave principle and either rewire the (pin = 0 case) or the in-links (pin = 1 case). The wave propagates either (A) laterally or (B) radially. (DOCX) [file pcbi.1011325.s004.docx]

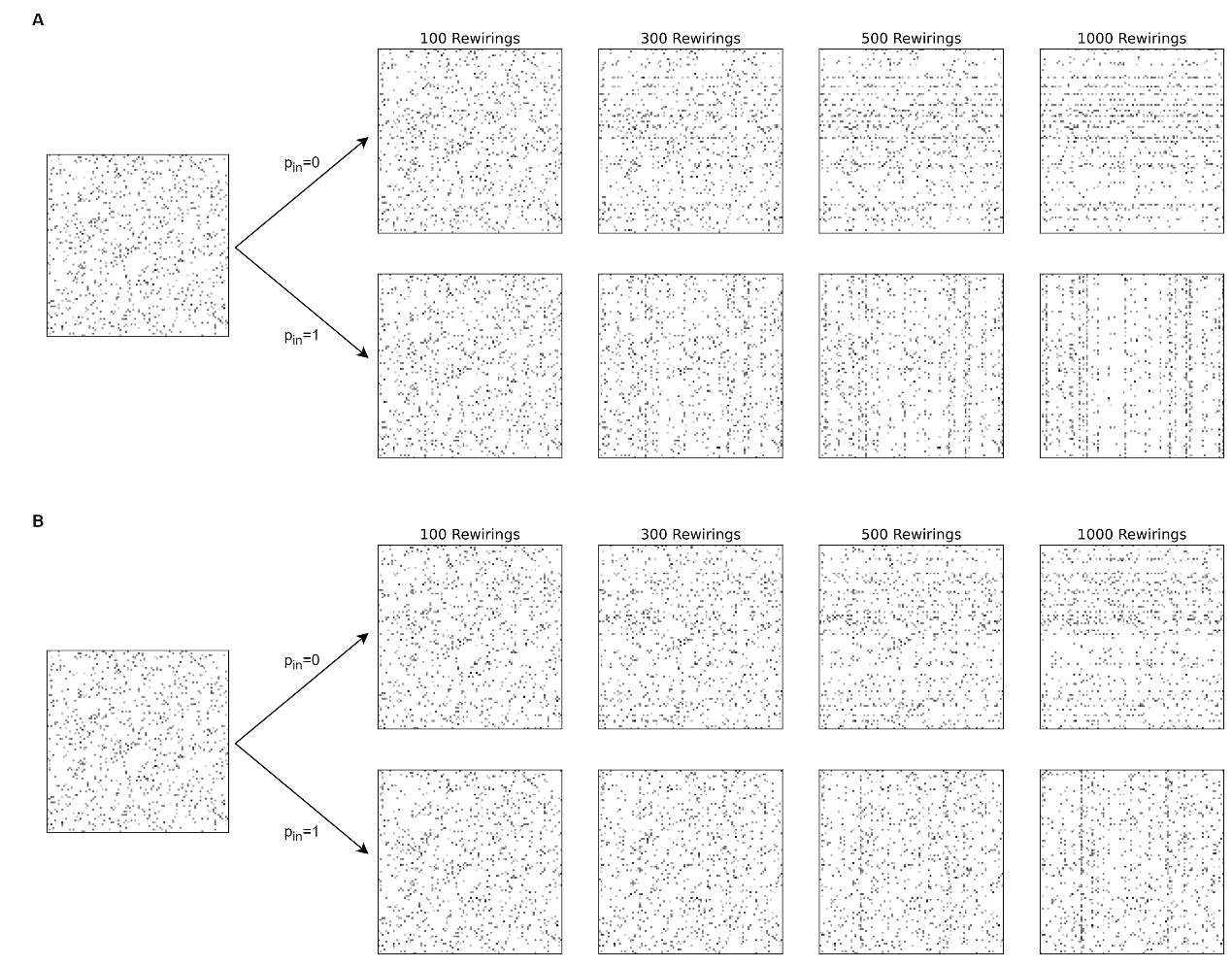


**Fig S4.** Applying the wave principle to the network starts forming hubs after 500 rewiring steps. Evolution of network connectivity when applying the wave principle and either rewire the (*p_in_* = 0 case) or the in-links (*p_in_* = 1 case). The wave propagates either (A) laterally or (B) radially.
