# Supplementary material for: Functional and spatial rewiring principles jointly regulate context-sensitive computation
Source: PLoS Comput Biol. 2023 Aug 11;19(8):e1011325. doi: 10.1371/journal.pcbi.1011325 (PMC10446201; doi:10.1371/journal.pcbi.1011325)
Supplement: S5 Fig — (A) The ‘functional + random algorithm and (B) the ‘functional + spatial’ algorithm without wave-based rewiring (pwave = 0), pin = 0.5 for both. (DOCX) [file pcbi.1011325.s005.docx]

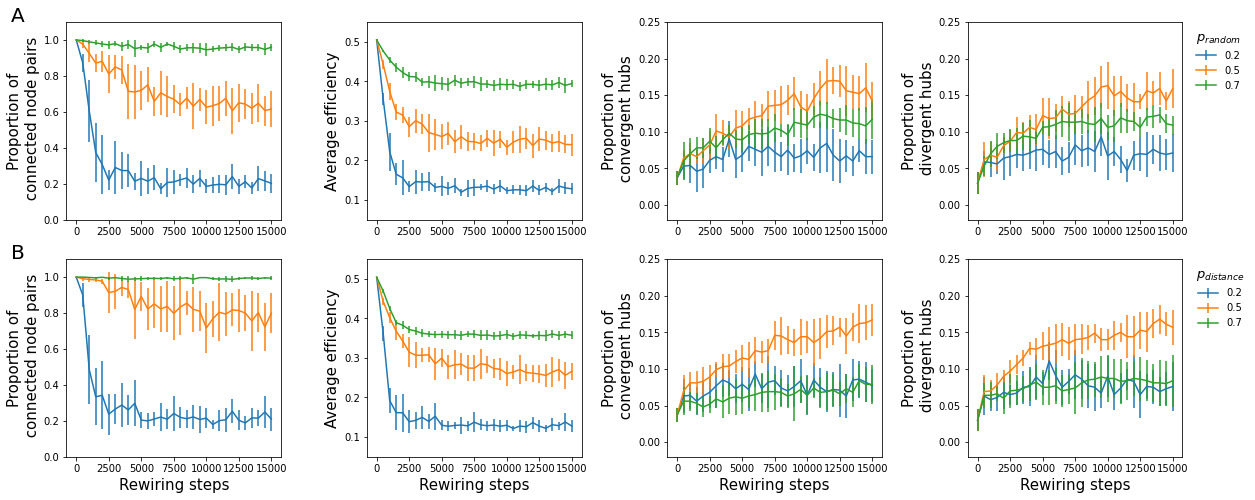


**Fig S5.** The proportion of connected node pairs, average efficiency, proportion of convergent and divergent hubs stabilize their values but never become completely static. (A) The ‘functional + random algorithm and (B) the ‘functional + spatial’ algorithm without wave-based rewiring ($p_{wave}=0$), $p_{in}=0.5$ for both.
