# Supplementary material for: Functional and spatial rewiring principles jointly regulate context-sensitive computation
Source: PLoS Comput Biol. 2023 Aug 11;19(8):e1011325. doi: 10.1371/journal.pcbi.1011325 (PMC10446201; doi:10.1371/journal.pcbi.1011325)
Supplement: S6 Fig — Average efficiency as a function of the proportion of adaptive rewiring, pfunction, pin, was set to 0.5. (DOCX) [file pcbi.1011325.s006.docx]

When a low proportion of adaptive rewiring is chosen ($p_{function}<0.4$) random rewiring yields higher efficiency than distance-based rewiring. When a small proportion of the distance-based rewiring is replaced with random rewiring, i.e., from $(p_{function},p_{distance})$ to $(p_{function},p_{distance}-0.1, p_{random}=0.1)$, average efficiency increases for $p_{function}<0.4$.


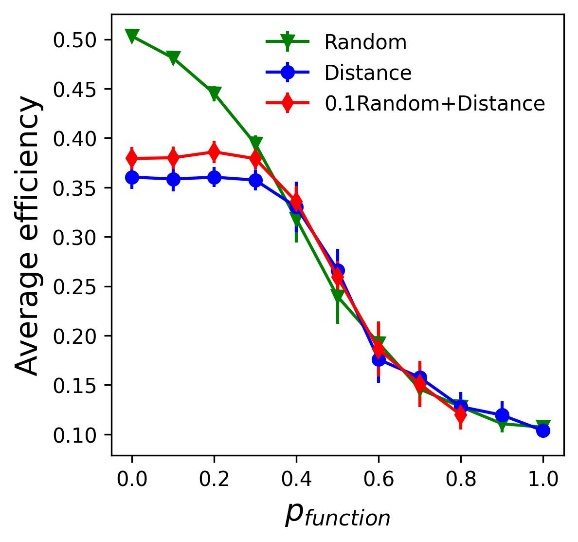


**Fig S6.** Including a small proportion of random rewiring into the combination of adaptive and distance-based rewiring can further increase average efficiency at low proportions of adaptive rewiring. Average efficiency as a function of the proportion of adaptive rewiring, $p_{function}$. $p_{in}$, was set to $0.5$.
