# Supplementary material for: Functional and spatial rewiring principles jointly regulate context-sensitive computation
Source: PLoS Comput Biol. 2023 Aug 11;19(8):e1011325. doi: 10.1371/journal.pcbi.1011325 (PMC10446201; doi:10.1371/journal.pcbi.1011325)
Supplement: S7 Fig — Modularity as a function of pdistance, for different probabilities of in-link rewiring, pin. (DOCX) [file pcbi.1011325.s007.docx]

Modularity measures the extent to which a network can be subdivided into communities. For the weighted directed graph, we use the following definition of modularity[1]:

$$\begin{aligned} Q=\frac{1}{2w}\sum_{i} \sum_{j} \left( w_{ij}-\frac{w_{i}^{in}w_{j}^{out}}{2w} \right)\delta\left( C_{i},Cj \right) \end{aligned}$$

In this equation, $w=\sum_{i} \sum_{j} w_{ij}$, $w_{i}^{in}=\sum_{j} w_{ij}$, $w_{j}^{out}=\sum_{i} w_{ij}$, $\delta(C_{i},C_{j})$ is the Kronecker delta function, and $C_{i}$ indicates the community that node $i$ belongs to. The communities are found by the algorithm proposed by Leicht and Newman[2].


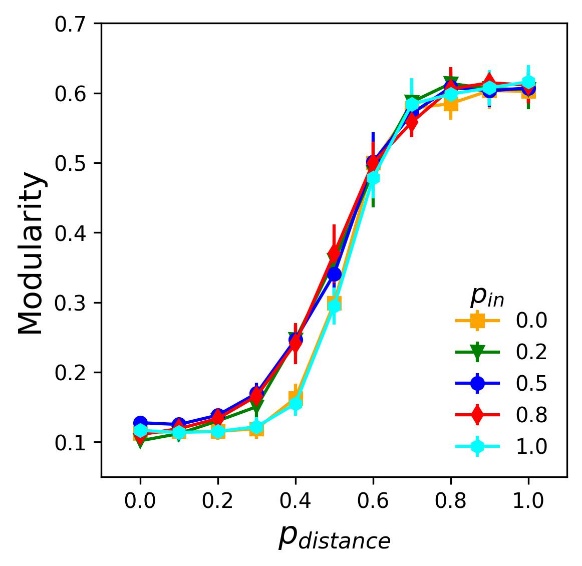


**Fig S7.** Modularity is proportional to the probability of distance-based rewiring, $p_{distance}$. Modularity as a function of $p_{distance}$, for different probabilities of in-link rewiring, $p_{in}$.

**References**

1. Arenas A, Duch J, Fernández A, Gómez S. Size reduction of complex networks preserving modularity. New J Phys. 2007;9: 176. doi:10.1088/1367-2630/9/6/176

2. Leicht EA, Newman MEJ. Community Structure in Directed Networks. Phys Rev Lett. 2008;100: 118703. doi:10.1103/PhysRevLett.100.118703
