# Supplementary material for: Functional and spatial rewiring principles jointly regulate context-sensitive computation
Source: PLoS Comput Biol. 2023 Aug 11;19(8):e1011325. doi: 10.1371/journal.pcbi.1011325 (PMC10446201; doi:10.1371/journal.pcbi.1011325)
Supplement: S8 Fig — Proportion of convergent and divergent hubs as a function of pdistance (A) for the lateral and (B) the radial field. (DOCX) [file pcbi.1011325.s008.docx]

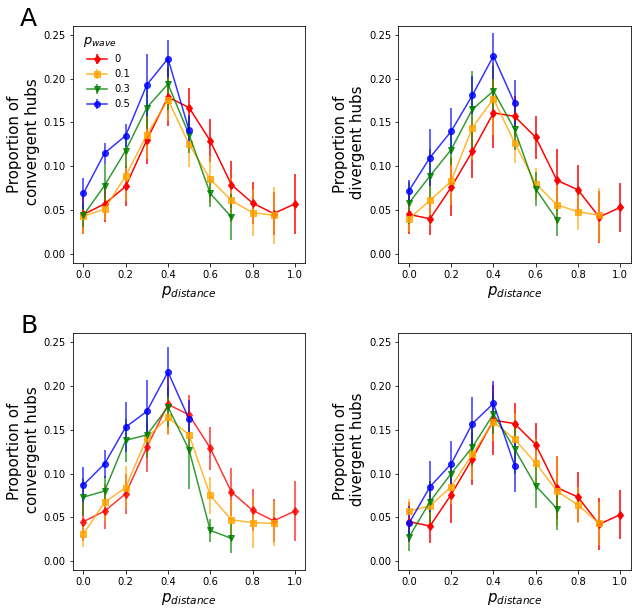


**Fig S8.** Rewiring based on the wave principle does not change the emergence of convergent and divergent hubs as a function of the proportion distance-based rewiring$.$ Proportion of convergent and divergent hubs as a function of $p_{distance}$ (A) for the lateral and (B) the radial field.
