# Supplementary material for: Functional and spatial rewiring principles jointly regulate context-sensitive computation
Source: PLoS Comput Biol. 2023 Aug 11;19(8):e1011325. doi: 10.1371/journal.pcbi.1011325 (PMC10446201; doi:10.1371/journal.pcbi.1011325)
Supplement: S9 Fig — The number of convergent-divergent units in rewired networks as a function of pdistance for the lateral and the radial field. (DOCX) [file pcbi.1011325.s009.docx]

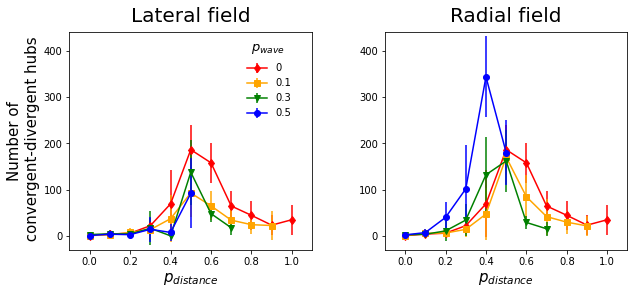


**Fig S9.** Wave-based rewiring reduces the number of convergent-divergent units in case of a lateral field, while increases it in case of a radial field. The number of convergent-divergent units in rewired networks as a function of $p_{distance}$ for the lateral and the radial field.
