# Supplementary material for: Functional and spatial rewiring principles jointly regulate context-sensitive computation
Source: PLoS Comput Biol. 2023 Aug 11;19(8):e1011325. doi: 10.1371/journal.pcbi.1011325 (PMC10446201; doi:10.1371/journal.pcbi.1011325)
Supplement: S10 Fig — Proportions of source and target nodes and their overlap with pdistance, for (A) the lateral and (B) radial field case. The dashed lines are for values when no wave-based rewiring is performed, i.e., pwave = 0. (DOCX) [file pcbi.1011325.s010.docx]

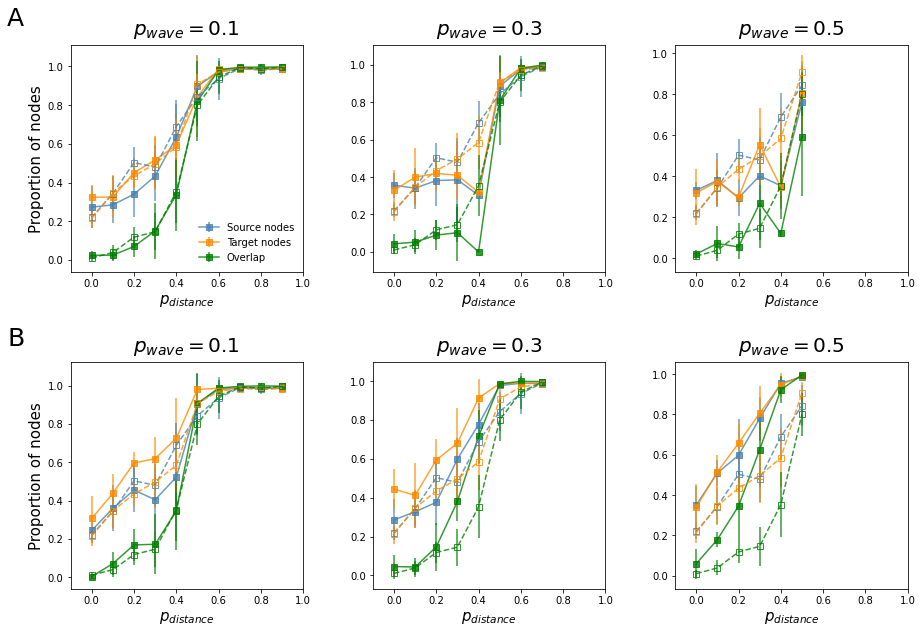


**Fig S10.** Wave-based rewiring dampens the proportion of source and target nodes and their overlap when $p_{wave}>0.1$ and $p_{distance}=0.4$ for the lateral field; it increases them when $p_{wave}>0.1$ for the radial field. Proportions of source and target nodes and their overlap with $p_{distance}$, for (A) the lateral and (B) radial field case. The dashed lines are for values when no wave-based rewiring is performed, i.e., $p_{wave}=0$.
