# Supplementary material for: Functional and spatial rewiring principles jointly regulate context-sensitive computation
Source: PLoS Comput Biol. 2023 Aug 11;19(8):e1011325. doi: 10.1371/journal.pcbi.1011325 (PMC10446201; doi:10.1371/journal.pcbi.1011325)
Supplement: S11 Fig — (A) Proportion of nodes in intermediate subgraphs and (B) density of intermediate subgraphs as a function of pdistance for the lateral and the radial field. (DOCX) [file pcbi.1011325.s011.docx]

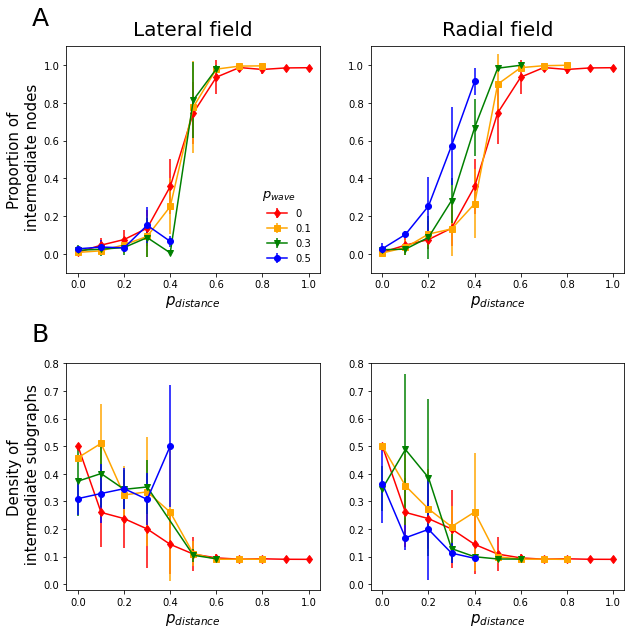


**Fig S11.** Wave-based rewiring reduces the size of intermediate subgraphs at $p_{distance}=0.4$ and $p_{wave}>0.1$, but increases the density of intermediate subgraphs when $p_{distance}>0$ for the lateral field; it increases the size of intermediate subgraphs when $p_{wave}>0.1$, but does not change the density of intermediate subgraphs in a systematic way for the radial field case. (A) Proportion of nodes in intermediate subgraphs and (B) density of intermediate subgraphs as a function of $p_{distance}$ for the lateral and the radial field.
