# Supplementary material for: Functional and spatial rewiring principles jointly regulate context-sensitive computation
Source: PLoS Comput Biol. 2023 Aug 11;19(8):e1011325. doi: 10.1371/journal.pcbi.1011325 (PMC10446201; doi:10.1371/journal.pcbi.1011325)
Supplement: S12 Fig — (A) The ‘functional + random’ algorithm and (B) the ‘functional + spatial’ algorithm without wave-based rewiring (pwave = 0), both for pin = 0.5. Dashed lines correspond to the results of the standard (deterministic) adaptive rewiring, and solid lines to stochastic adaptive rewiring. (DOCX) [file pcbi.1011325.s012.docx]

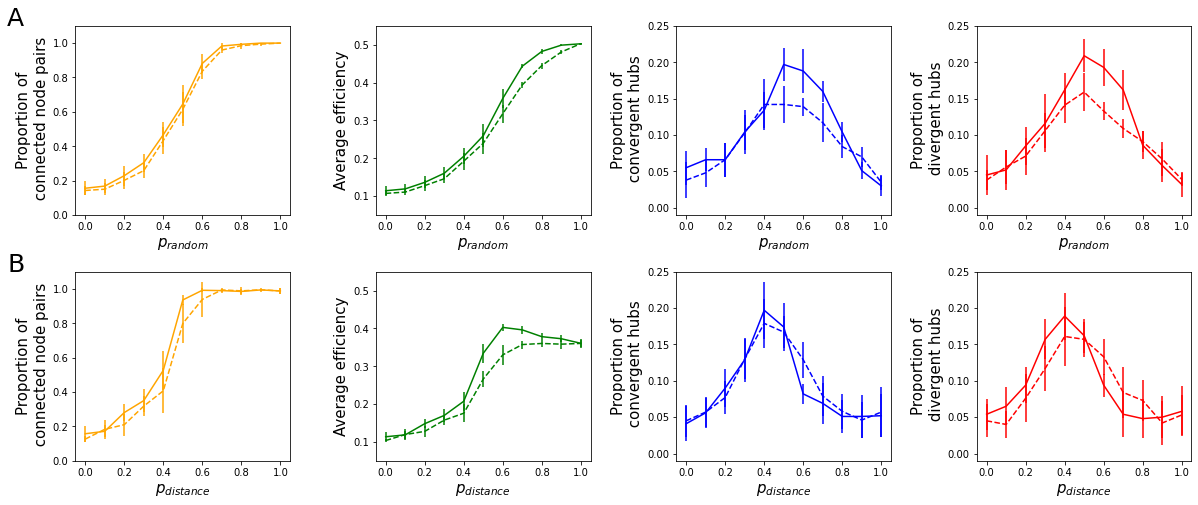


**Fig S12.** Using stochastic instead of the standard (deterministic) adaptive rewiring does not change the way $p_{random}$ and $p_{distance}$ control the proportion of connected node pairs, average efficiency, and the proportion of convergent and divergent hubs. (A) The ‘functional + random’ algorithm and (B) the ‘functional + spatial’ algorithm without wave-based rewiring ($p_{wave}=0$), both for $p_{in}=0.5$. Dashed lines correspond to the results of the standard (deterministic) adaptive rewiring, and solid lines to stochastic adaptive rewiring.
