# Supplementary material for: Functional and spatial rewiring principles jointly regulate context-sensitive computation
Source: PLoS Comput Biol. 2023 Aug 11;19(8):e1011325. doi: 10.1371/journal.pcbi.1011325 (PMC10446201; doi:10.1371/journal.pcbi.1011325)
Supplement: S13 Fig — (A) The combination of stochastic adaptive rewiring and random rewiring. (B) The combination of stochastic adaptive rewiring and distance-based rewiring. pin is 0.5 for both cases. (DOCX) [file pcbi.1011325.s013.docx]

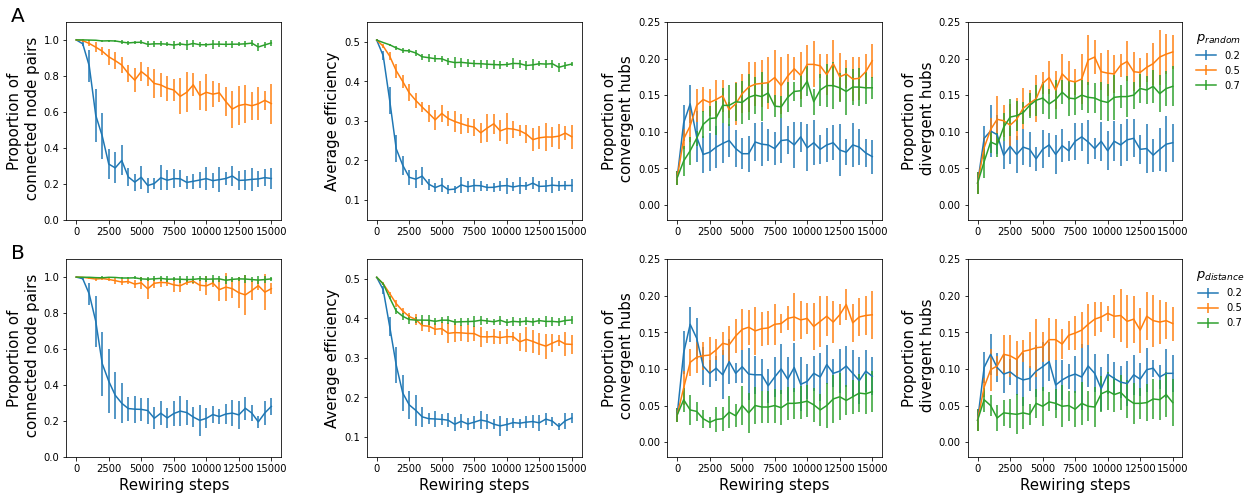


**Fig S13.** The proportion of connected node pairs, average efficiency, convergent and divergent hubs stabilize but are never completely static. (A) The combination of stochastic adaptive rewiring and random rewiring. (B) The combination of stochastic adaptive rewiring and distance-based rewiring. $p_{in}$ is 0.5 for both cases.
