# Supplementary material for: Functional and spatial rewiring principles jointly regulate context-sensitive computation
Source: PLoS Comput Biol. 2023 Aug 11;19(8):e1011325. doi: 10.1371/journal.pcbi.1011325 (PMC10446201; doi:10.1371/journal.pcbi.1011325)
Supplement: S14 Fig — (A) Combination of stochastic adaptive rewiring and random rewiring, and (B) combination of stochastic adaptive rewiring and distance-based rewiring, both for pin = 0.5. Dashed lines correspond to the results of the standard (deterministic) adaptive rewiring, solid lines to the stochastic adaptive rewiring. (DOCX) [file pcbi.1011325.s014.docx]

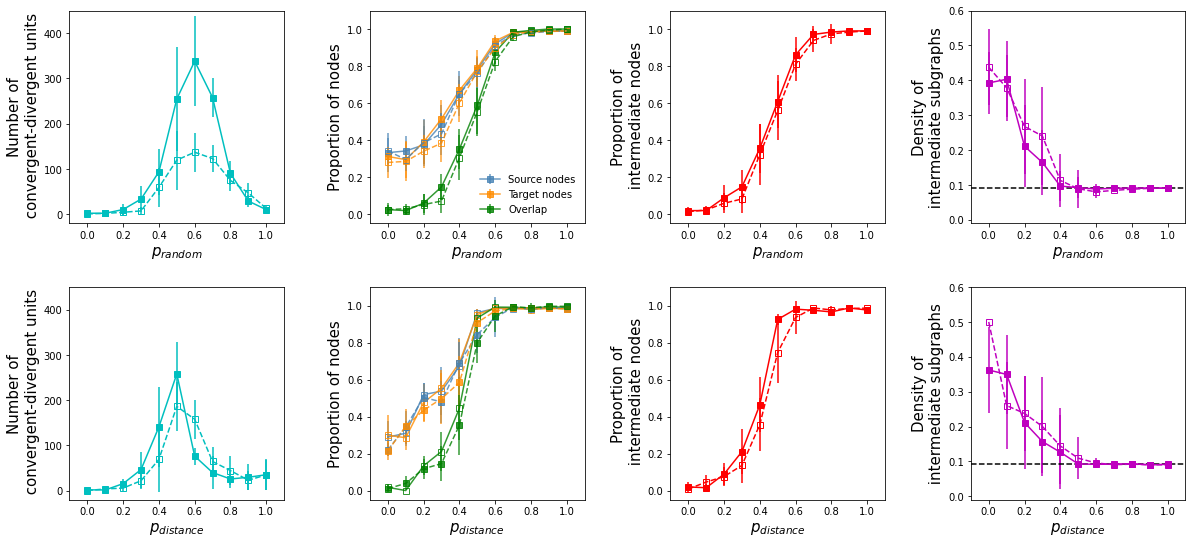


**Fig S14.** Using stochastic adaptive rewiring instead of deterministic increases the number of convergent-divergent units. (A) Combination of stochastic adaptive rewiring and random rewiring, and (B) combination of stochastic adaptive rewiring and distance-based rewiring, both for $p_{in}=0.5$. Dashed lines correspond to the results of the standard (deterministic) adaptive rewiring, solid lines to the stochastic adaptive rewiring.
